# Prevalence and Resistance Profiles of Pediatric Enterococcal Isolates: A Five-Year Update from Children's Medical Center Hospital, Tehran

**DOI:** 10.1155/2024/5529598

**Published:** 2024-07-30

**Authors:** Bahram Nikmanesh, Sajjad Yazdansetad, Mona Konkori, Mehrzad Sadredinamin, Zohreh Ghalavand, Neda Yousefi Nojookambari

**Affiliations:** ^1^ Department of Medical Laboratory Sciences School of Allied Medical Sciences Tehran University of Medical Sciences, Tehran, Iran; ^2^ Department of Biology Faculty of Basic Sciences Imam Hossein Comprehensive University, Tehran, Iran; ^3^ Department of Medical Laboratory Children's Medical Center Hospital, Tehran, Iran; ^4^ Department of Microbiology School of Medicine Shahid Beheshti University of Medical Sciences, Tehran, Iran

## Abstract

**Background:**

In this study, attempts were made to evaluate the frequency of high-level gentamicin-resistant (HLGR) and vancomycin-resistant enterococci (VRE) and the prevalence and antibiotic resistance profile of enterococcal species isolated from pediatric patients referred to Children's Medical Center Hospital, Tehran, over five years.

**Materials and Methods:**

A total of 404 enterococcal isolates from different patients referred to the Children's Medical Center between March 2016 and March 2021 were included in this cross-sectional study. Antimicrobial susceptibility testing was performed using standard methods according to the guidelines of the Clinical Laboratories Standards Institute (CLSI).

**Results:**

Approximately one-third of the enterococcal strains were isolated from urology and intensive care units. 17.3% of the isolates were obtained from outpatient sources. However, 82.7% of the isolates were sourced from inpatient settings. We found that the rates of resistance to ampicillin, penicillin, and vancomycin were twice as high in inpatients as in outpatients. Of the total isolates, 87.4% and 49.3% were identified as HLGR and VRE, respectively. In addition, we identified 2% of the VRE isolates that were not susceptible to linezolid. Nitrofurantoin showed excellent activity against enterococcal isolates in the urine, with a susceptibility rate of 92.5%.

**Conclusion:**

The present study reports the highest range of VRE isolated from pediatric patients in Iran. Despite the predominance of HLGR enterococci in our region, vancomycin remains effective against such strains. This study is among the few to demonstrate the incidence of linezolid-insensitive VRE in pediatric patients. Therefore, it is important to evaluate effective infection control measures to prevent linezolid and vancomycin resistance in enterococci.

## 1. Introduction

*Enterococcus* species, particularly *Enterococcus faecalis* and *Enterococcus faecium*, are common opportunistic bacteria found in the human gastrointestinal (GI) tract, vagina, and oral cavity. They can cause severe infections in individuals under specific conditions [1]. Any disruption in the host/commensal balance that weakens the host defense system or environmental factors, such as the use of antibiotics that inadvertently promote the growth of resistant enterococci, can lead to life-threatening enterococcal infections [2]. In recent decades, enterococci have become significant nosocomial pathogens and are frequently isolated from serious hospital-acquired infections, including infective endocarditis (IE); urinary tract infections (UTIs); bacteremia; nosocomial meningitis; and intraabdominal, wound, and pelvic infections [3].

Enterococci, although not typically highly virulent, pose a significant challenge in clinical settings due to their high resistance to antimicrobial agents [4]. The intrinsic resistance of these bacteria, along with their ability to acquire additional resistance mechanisms and to thrive in hospital environments, complicates the treatment of enterococcal infections [5]. The rise of high-level aminoglycoside resistance (HLAR) and vancomycin-resistant enterococci (VRE) in clinical isolates is a growing global concern, leading to more severe infections and posing serious health risks [6, 7]. The increasing prevalence of HLAR and VRE isolates in areas such as Iran has alerted healthcare professionals, requiring strict infection control measures and presenting complex treatment challenges for clinicians. Actions to combat the spread of resistant enterococci strains are essential for maintaining public health and ensuring effective patient care [8–11].

The prevalence and impact of drug-resistant enterococcal infections among pediatric patients remain inadequately understood, despite extensive research in adult populations. This knowledge gap underscores the importance of investigating the epidemiology and antibiotic resistance profiles of enterococcal isolates in children. The current study sought to address this gap by evaluating the prevalence and resistance patterns of enterococcal infections in pediatric populations for five years.

## 2. Materials and Methods

### 2.1. Patients and Clinical Specimens

The present cross-sectional study was carried out on children with any obvious enterococcal infection referred to the Children's Medical Center Hospital in Tehran from March 2016 to March 2021. Various clinical samples were collected from outpatients and inpatients (hospitalized patients). In our study, UTI was defined as an infection of the urinary tract with 10^4^ CFU of cultured enterococci in urine and at least one UTI symptom, such as fever or urinary frequency. Only one isolate per patient was included in the study. The patients' personal and clinical information was recorded, including age, sex, length of hospital stay, time and ward of strain isolation, and microbiological data.

### 2.2. Identification of Enterococcal Isolates

Standard conventional biochemical tests for the identification of isolates were performed on colonies from the primary cultures. For this purpose, all suspected colonies of *Enterococcus* spp. were examined using Gram staining and further identified using the catalase test, hemolysis examination, bile-esculin agar, and growth in 6.5% NaCl. The isolates were stored at −70°C in trypticase soy broth containing 10% glycerol for later analysis.

### 2.3. Detection of HLGR and VRE Isolates

HLGR was detected using an agar-screening method. In brief, 10 *μ*L of bacterial suspension adjusting to 0.5 McFarland turbidity standard was spotted on a brain-heart infusion (BHI) agar plate (HiMedia, India) containing 500 *μ*g/mL gentamicin. The plates were incubated at 35 ± 2°C for 24 h. The growth of more than one colony in the spotted zone was considered HLGR. The control quality of the culture plates was determined by reincubation at 35 ± 2°C for 24 h. VRE isolates were also detected by culturing on BHI agar supplemented with 6 *μ*g/mL vancomycin (HiMedia, Mumbai, India), according to a previously described method [10, 12].

### 2.4. Antimicrobial Susceptibility Testing

Antibiotic susceptibility tests were performed using the standardized Kirby–Bauer disc diffusion technique on Mueller–Hinton agar (HiMedia, Mumbai, India). Commercially available disks of antimicrobial drugs (Mast Group, UK), which have been most frequently used for the treatment of enterococcal infections, were selected and tested as recommended by the Clinical and Laboratory Standards Institute [13] (“CLSI (2021) Performance Standards for Antimicrobial Susceptibility Testing; Twenty-Fifth Informational Supplement; Clinical and Laboratory Standards Institute, Wayne, M100-S25.,” n.d.). Accordingly, the susceptibility of the isolates to the following antibiotics was examined: penicillin G (10 units/disk), ampicillin (10 *μ*g/disk), ampicillin-sulbactam (10 + 10 *μ*g/disk), gentamicin (10 *μ*g/disk), nitrofurantoin (300 *μ*g/disk) (only for patients with a suspected UTI), and linezolid (30 *μ*g/disk). Susceptibility to vancomycin was confirmed by determining the MIC using Epsilometer (E) test strips (Liofilchem®, Italy). *E. faecalis* ATCC 29212 was used as a quality control reference strain.

### 2.5. Statistical Analysis

Data analysis was performed using Excel 2022 and IBM SPSS Statistics version 20.0 (Chicago, IL, USA).

## 3. Results

A total of 404 clinical isolates of *Enterococcus* spp. were obtained from children over five years. The patients' ages ranged from infancy (1 day) to 15 years. The median patient age was 3 ± 1 months. 47.5% of the patients were female, and 52.5% were male.

The proportions of enterococcal isolates from 2016 to 2021 were 71.3% (288 out of 404), 25.5% (103 out of 404), and 3.2% (13 out of 404) for *Enterococcus faecium*, *Enterococcus faecalis*, and *Enterococcus* spp., respectively.

The distribution of enterococci isolates based on the inpatient and outpatient clinics is outlined in Table 1. The data reveal that 17.3% (70 out of 404) of the isolates originated from outpatients, whereas the vast majority, constituting 82.7% (334 out of 404), were sourced from inpatients.

Seventy out of 404 isolates (17.3%) were collected from outpatients or those referred to emergency department and classified as community-acquired enterococci and the remainder (82.7%) from different wards as follows: urology (10.4%), pediatric intensive care unit (PICU) (7.4%), respiratory (10%), nephrology and dialysis (9.6%), neonatal intensive care unit (NICU) (4.8%), surgical (3.9%), neonatal (3.9%), gastroenterology (2.9%), infection (1.9%), neurology (1.4%), and oncology (1.4%), were considered as nosocomial enterococci.

A significant proportion of isolates was recovered from urine samples (55.9%, *n* = 226), followed by blood culture (29.7%, *n* = 120), wounds (3.5%, *n* = 14), nasal discharge (1.7%, *n* = 7), bronchoalveolar lavage (BAL) (1.2%, *n* = 5), ascitic fluid (1%, *n* = 4), abscess (0.7%, *n* = 3), CSF (0.7%, *n* = 3), dialysis fluid (0.5%, *n* = 2), peritoneal cavity (0.5%, *n* = 2), synovial fluid (0.5%, *n* = 2), central venous line (CVL) (0.2%, *n* = 1), urinary catheter (0.2%, *n* = 1), and other body fluid samples (3.5%, *n* = 14).

In our study, all isolates were assessed for resistance rates without species differentiation (Table 2), revealing high-level gentamicin resistance (HLGR) at 87.4% (90.9% in *E. faecalis* and 87.7% in *E. faecium*) and ciprofloxacin resistance at 98.2% (84.6% in *E. faecalis* and 99.3% in *E. faecium*). Ampicillin resistance rate was 60.9% (14.6% in *E. faecalis* and 78.8% in *E. faecium*), while vancomycin resistance was 49% (11.7% in *E. faecalis* and 63.5% in *E. faecium*) (Table 3). All the isolates were resistant to ampicillin-sulbactam. The resistance rates of ampicillin, penicillin, and vancomycin in inpatients were twice as high as those in outpatients (Table 4). 98% of the isolates were susceptible to linezolid and 2% were resistant (0% in *E. faecalis* and 2.2% in *E. faecium*). 92.5% of the isolates were susceptible and 5.3% were resistant to nitrofurantoin (0% in *E. faecalis* and 7.9% in *E. faecium*).

**Figure 1:**
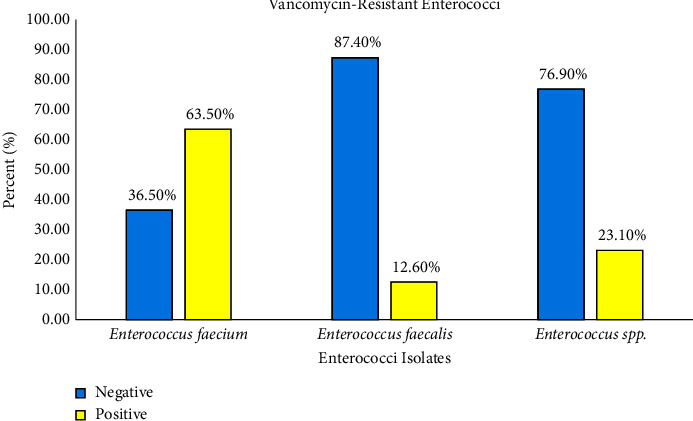
A descriptive panel of VRE and non-VRE *Enterococcus* strains.

The antibiotic susceptibility results showed that linezolid was the most active antibiotic against the tested enterococcal isolates (98% susceptibility rate). Nitrofurantoin showed excellent activity against urinary enterococcal isolates (92.5% as the susceptibility rate). In addition, 49.3% (*n* = 199) of the isolates were identified to have the VRE phenotype (12.6% in *E. faecalis* and 63.5% in *E. faecium*). As well as VRE phenotype was seen in 56.9% of inpatients and 12.9% of outpatients.  Figure 1 shows a panel of VRE and non-VRE *Enterococcus* strains.

Forty-two point one percent (42.1%) of the total enterococci isolates were MDR (11.7% in *E. faecalis* and 54.2% in *E. faecium*). MDR enterococci isolates were detected in 48.8% of inpatients and 10% of outpatients.

**Figure 2:**
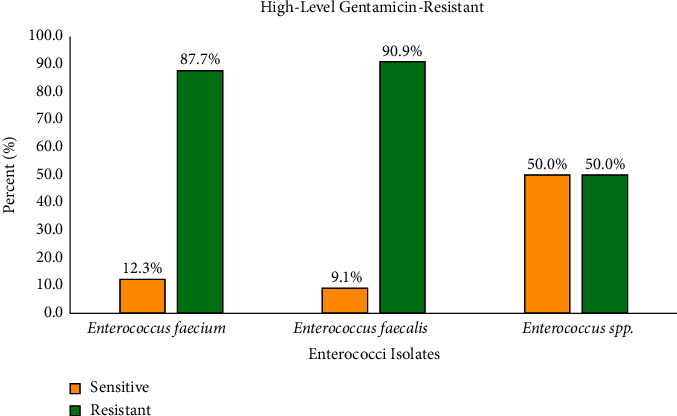
A descriptive panel of HLGR and non-HLGR *Enterococcus* strains.

The HLGR phenotype was detected in 87.4% of *E. faecalis*, *E. faecium*, and other *Enterococcus* spp. isolates. The majority (90.9%) of HLGR was found in *E. faecalis* isolates.  Figure 2 displays a panel of HLGR and non-HLGR *Enterococcus* strains.

Altogether, five of 404 (1.2%) enterococcal isolates had both VRE and HLGR phenotypes and were resistant to linezolid. In addition, resistance to linezolid and nitrofurantoin was only observed in *E. faecium*, and a greater number of *E. faecium* strains showed resistance to other antibiotics than *E. faecalis* strains.

## 4. Discussion

Since the late twentieth century, when enterococci have emerged as a significant cause of nosocomial infections, bacteria have become the second most common nosocomial pathogen and the third leading cause of nosocomial bloodstream infections [14]. Enterococcal infections can include serious life-threatening disorders, especially in children, such as meningitis, septicemia, and infective endocarditis [15]. However, few studies have been conducted on enterococci isolated from children in Iran. In our study, *E. faecium* and *E. faecalis* comprised 71.3 and 25.5% of the *Enterococcus* spp. isolated from pediatric patients, respectively. This observation is similar to reports from other regions of Iran and other countries, in which the distribution of enterococcal species derived from clinical samples, such as blood, urine, pleural fluid, cerebrospinal fluid, sputum, ascites, and hydrothorax, changed in favor of *E. faecium* [16–19]. In contrast, some studies have shown that the frequency of *E. faecalis* isolates in clinical specimens is usually two to three times higher than that of *E. faecium* isolates [20, 21]. This difference may be due to the different origins of the collected samples and different geographical regions. Furthermore, the increase in the prevalence of *E. faecium* species may be due to the common resistance of enterococci to antienterococcal drugs such as ampicillin, aminoglycosides, and glycopeptides [22, 23]. In our study, the highest vancomycin resistance was found in *E. faecium* isolates. The occurrence of VRE diverges in different countries, with a high frequency described in VRE in the US, the UK, Saudi Arabia, Ireland, and Turkey, whereas a low percentage is specific to some European countries such as Italy and France [24, 25].

Invasive enterococcal infections pose a significant challenge due to the emergence of drug-resistant strains. The standard treatment of vancomycin, ampicillin, and aminoglycoside antibiotics is becoming less effective as resistance to these antibiotics grows [26]. Vancomycin-resistant *Enterococcus* (VRE) and high-level gentamicin-resistant (HLGR) strains are particularly concerning, as they are responsible for a rising number of nosocomial infections in both adults and children [27]. Identifying alternative treatment options for these drug-resistant enterococci is crucial for the effective management and treatment of such infections [28]. Little data are available on the epidemiology and impact of VRE and HLGR infections in Iranian children. In this survey, we investigated the epidemiology and prevalence of clinical isolates of enterococci among the pediatric patients during the five years. Approximately one-third of the enterococcal strains were isolated from urology and PICU wards. Previously, a similar distribution was reported among hospitalized children in the urology ward located at the Children's Medical Center by Pourakbari et al.[29] and Sabouni et al. [30]. The high frequency of enterococcal strains isolated from the urology ward demands more attention to infection prevention and control (IPC) programs than in other units [31].

Enterococci are typically antibiotic-resistant. Owing to the intrinsic and acquired resistance to most antibiotics, the addition of vancomycin resistance meant that many enterococcal infections have become untreatable [32]. The high isolation rate (49.3%) of clinical VRE in our study raised significant concerns. It was found that the majority of VRE isolates were obtained from inpatients, with a lower percentage originating from outpatients. However, the prevalence of VRE in a specific population can vary significantly due to factors such as geographical location, duration of hospital exposure, usage of medical devices, sample size, and others [33].

To date, this is the largest high-risk pediatric population screened for VRE infections. A lower distribution of VRE (16%) was observed in another study conducted in children at the Children's Medical Center [29]. In this study, *E. faecium* was the predominant VRE isolate (45.30%), which is consistent with the results of similar studies [34, 35]. In contrast, *E. faecalis* is the predominant VRE, which has been reported in other studies [36]. Based on these findings, a substantial increase in the hospitalization rate for VRE infection among pediatric patients has been observed over the last few years, paralleling the increase observed in the hospitalized adult population [37]. The reason for this increase in VRE incidence may be the amalgamation of the same factors, including selective pressure from broad-spectrum antimicrobial agents, an increasing number of critically ill and immunosuppressed at-risk patients, and increased nosocomial transmission owing to deficiencies in hospital infection control practices [38]. In addition, it has been reported that long-term use of vancomycin and cephalosporins can potentially lead to the selection of intestinal carriage of vancomycin-resistant enterococci (VRE) [39]. It is important to remember that VRE infections are associated with high morbidity and mortality rates as well as increased duration and cost of hospitalization [40]. Owing to the excellent performance of hospital infection control and accuracy in prescribing antibiotics in developed countries, a very low prevalence of VRE has been reported in countries such as Japan and the United States [37, 41]. The majority of isolates showed more than 80 percent resistance to high levels of gentamicin *in vitro*. In numerous studies conducted both in Iran and other countries, a high prevalence of HLGR has been reported in *E. faecalis* [42, 43]. This may be attributed to its higher prevalence in clinical settings. However, our report deviates from this trend, as we observed a higher frequency of HLGR in *E. faecium*, aligning with the findings of Hayakawa et al. [36]. This divergence highlights the need for further research to understand the varying patterns of HLGR in different *Enterococcus* species. As well as, it is necessary to consider the origin of the clinic (inpatient or outpatient) when assessing resistance rates. Our study revealed that HLGR was significantly more common among inpatients compared to outpatients. This disparity in resistance rates among *E. faecium* isolates could potentially be attributed to this difference in clinical settings [19].

Overall, the isolation rate of HLGR was greater than that of VRE in our study, which is similar to the findings of other studies [44]. The treatment of HLGR enterococci poses a significant challenge in healthcare settings. Recent reports have suggested the use of an antipeptidoglycan active agent in combination with a membrane-active agent to address this problem [45]. Despite the predominance of HLGR enterococci in our region, vancomycin continues to demonstrate antimicrobial efficacy against such strains. However, the emergence of VRE isolates is a cause for concern and warrants continuous surveillance using both genetic and phenotypic methods to address this type of resistance. This “red alarm” highlights the importance of proactive measures in our health system to effectively manage and mitigate the impact of HLGR enterococci [46].

In our surveillance, a high prevalence of multidrug-resistant (MDR) enterococcal isolates, defined as resistance to three or more antibiotic classes, was observed among pediatric patients. The prevalence of MDR enterococci was more common in *E. faecium* (54.2%) compared to *E. faecalis* (11.7%), which is in concordance with previous studies [33, 47]. In addition, the MDR phenotypes were significantly higher for both *E. faecium* and *E. faecium* of inpatient isolates compared to those from outpatients.

It has been shown that the majority of VRE isolates had additional resistance to penicillin G, ampicillin, ampicillin-sulbactam, and gentamicin. However, the resistance to these antibiotics was significantly lower than that in the non-VRE isolates. Generally, *E. faecium* is less susceptible to *β*-lactam agents than *E. faecalis* because their penicillin-binding proteins (PBPs) have lower affinities for these antibiotics [48]. In addition, resistance mechanisms to other antibacterial agents, except *β*-lactams, could be encoded by conjugative transposons, and the presence of MDR isolates may facilitate the increase, transmission, and spread of drug resistance in hospital wards [49]. Previously, clinical isolates of MDR enterococci have been frequently reported in several studies. In developing countries, unreasonable administration of antimicrobial agents, especially beta-lactam antibiotics, may be responsible for developing MDR enterococci [47].

Linezolid is used as the first-line treatment for infections caused by VRE. The potential coexistence of linezolid and vancomycin resistance among enterococcal strains also raises concerns, considering the importance of these drugs in the treatment of MDR enterococcal isolates [50].

In the present study, the rate of linezolid resistance was 2%, whereas various studies have reported 100% sensitivity to linezolid [51]. An intriguing aspect of this study was the identification of four VRE strains from pediatric patients who were not susceptible to linezolid. Our results were similar to those of a recent study that found four clinical isolates of *E. faecium* that were resistant to both linezolid and vancomycin [52].

Limited treatment options are available for linezolid resistance VRE (LRVRE) with difficult-to-treat infections. Current treatment options such as linezolid, daptomycin, quinupristin/dalfopristin, and tigecycline have shown promising efficacy against VRE infections [53]. Moreover, combination therapy may offer a distinct advantage over monotherapies due to their broad spectrum and synergistic effect [54]. The use of quinupristin/dalfopristin (Q/D) in combination with rifampin or doxycycline and daptomycin and/or linezolid in combination with ampicillin, tigecycline, doxycycline, rifampin, and fluoroquinolones has been reported in some literature for the treatment of VRE infections [55].

Although molecular typing was not performed in this study to determine the potential clonality of the linezolid-nonsusceptible VRE strain from the same region, this was a possibility, as the nosocomial spread of linezolid- and vancomycin-resistant enterococci has been previously described [56].

While linezolid nonsusceptibility remains sporadic among enterococci, constant surveillance of linezolid activity is required to control these opportunistic pathogens.

## 5. Conclusion

To our knowledge, this study is among the few to show the prevalence of VRE and linezolid nonsusceptible enterococcal isolates in pediatric patients. Therefore, efficient and accurate epidemiological typing methods such as pulsed-field gel electrophoresis (PFGE) have been suggested for surveillance and to limit the occurrence and spread of epidemic clones within and between hospitals and community settings. Our study showed a rise in resistance to gentamicin and vancomycin, highlighting the need for a reassessment of antibiotic treatments in Iran. Moreover, to prevent the spread of VRE and linezolid nonsusceptible strains in healthcare settings, resistance measures derived from those used in many hospitals were implemented. Consequently, antimicrobial susceptibility testing is essential for managing enterococcal infections, and antibiotics should be prescribed carefully. Continuous monitoring of HLGR and VRE prevalence and drug susceptibilities for both inpatient and community isolates is highly recommended.

## Figures and Tables

**Table 1:** Distribution of enterococci isolates in inpatient and outpatient clinics.

Inpatient/outpatient clinics	No. (%) of *Enterococcus faecium*	No. (%) of *Enterococcus feacalis*	No. (%) of *Enterococcus* spp.	No. (%) of total isolates
Inpatient	258 (77.24%)	67 (20%)	9 (2.69%)	334 (82.67%)
Outpatient	30 (42.85%)	36 (51.42%)	4 (5.71%)	70 (20.95%)
Total clinics	288	103	13	404

**Table 2:** Antibiotic susceptibility testing of the enterococci isolates without species differentiation.

Antibiotic	Sensitive	Intermediate	Resistance (%)
Ampicillin	38.9%	2%	60.9
Penicillin	36.5%	0.7%	62.8
Ampicillin-sulbactam	0	0	100
Vancomycin	50.2%	0.7%	49
Gentamycin	12.5%	0	87.4
Ciprofloxacin	6%	1.2%	98.2
Linezolid	98%	0	2
Nitrofurantoin	92.5%	2.2%	5.3

**Table 3:** Antibiotic susceptibility testing of the enterococci isolates with species differentiation.

Antibiotic	*Enterococcus faecium*			*Enterococcus feacalis*			*Enterococcus* spp.		
	Sensitive	Intermediate	Resistance (%)	Sensitive	Intermediate	Resistance	Sensitive	Intermediate	Resistance
Ampicillin	20.8%	0.3%	78.8	85.4%	0	14.6%	69.2%	0	30.8%
Penicillin	18.1%	0.7%	81.2	84.5%	1	14.6%	61.5%	0	38.8%
Ampicillin-sulbactam	0	0	100	0	0	100%	0	0	100%
Vancomycin	36.5%	0	63.5	86.4%	1.9%	11.7%	69.2%	7.7%	23.1%
Gentamycin	12.3%	0	87.7	9.1%	0	90.9%	50%	0	50%
Ciprofloxacin	0%	0.7%	99.3	7.7	7.7%	84.6%	0	0	100%
Linezolid	97.8%	0	2.2	100%	0	0	100%	0	0
Nitrofurantoin	89.4%	2.6%	7.9	98.6%	0	1.4%	100%	0	0

**Table 4:** Antibiotic susceptibility testing of *Enterococcus* isolates according to their clinical source.

Antibiotic	Inpatients			Outpatients		
	Sensitive	Intermediate	Resistance (%)	Sensitive	Intermediate	Resistance
Ampicillin	31.73%	0.2%	67.96	72.85%	0	27.14%
Penicillin	29.34%	0.89%	69.76	70%	0	30%
Ampicillin-sulbactam	0	0	100	0	0	100%
Vancomycin	42.8%	0.59%	54.7	85%	1.4%	12.28%
Gentamycin	12.5%	0	87.5	14.2%	0	85.71%
Ciprofloxacin	0.63%	0.63%	98.72	0	12.5%	87.5%
Linezolid	97.07%	0	2.1	100%	0	0
Nitrofurantoin	92.07%	1.82%	6.09	93.65%	3.17%	3.17%

## Data Availability

The data used to support the findings of this study are available from the corresponding author upon request.

## References

[1] Krawczyk B., Wityk P., Gałęcka M., Michalik M. (2021). The many faces of Enterococcus spp.—commensal, probiotic and opportunistic pathogen. *Microorganisms*.

[2] Ramos S., Silva V., Igrejas G., Poeta P. (2020). Enterococci, from harmless bacteria to a pathogen. *Microorganisms*.

[3] Guzman Prieto A. M., van Schaik W., Rogers M. R. C. (2016). Global emergence and dissemination of enterococci as nosocomial pathogens: attack of the clones?. *Frontiers in Microbiology*.

[4] Gold H. S. (2001). Vancomycin-resistant enterococci: mechanisms and clinical observations. *Clinical Infectious Diseases*.

[5] Shepard B. D., Gilmore M. S. (2002). Antibiotic-resistant enterococci: the mechanisms and dynamics of drug introduction and resistance. *Microbes and Infection*.

[6] Bhatt P., Patel A., Sahni A. (2015). Emergence of multidrug resistant enterococci at a tertiary care centre. *Medical Journal Armed Forces India*.

[7] Haghi F., Lohrasbi V., Zeighami H. (2019). High incidence of virulence determinants, aminoglycoside and vancomycin resistance in enterococci isolated from hospitalized patients in northwest Iran. *BMC Infectious Diseases*.

[8] Moghimbeigi A., Moghimbeygi M., Dousti M. (2018). Prevalence of vancomycin resistance among isolates of enterococci in iran: a systematic review and meta-analysis. *Adolescent Health, Medicine and Therapeutics*.

[9] Mousavi S. H., Peeri-Doghaheh H., Mohammadi-Ghalehbin B. (2020). High-level resistance to aminoglycosides and ampicillin among clinical isolates of enterococcus species in an Iranian Referral Hospital. *Iranian Journal of Microbiology*.

[10] Jannati E., Amirmozaffari N., Saadatmand S., Arzanlou M. (2020). Faecal carriage of high-level aminoglycoside-resistant and ampicillin-resistant Enterococcus Species in Healthy Iranian Children. *Journal of Global Antimicrobial Resistance*.

[11] Jannati E., Khademi F., Manouchehrifar M. (2023). Antibiotic resistance and virulence potentials of E. faecalis and *E. faecium* in hospital wastewater: a case study in Ardabil, Iran. *Journal of Water and Health*.

[12] Li X. S., Qi Y., Li P. H. (2023). Genetic characterization of MDR genomic elements carrying two *aac(6′)-aph(2″)* genes in feline-derived clinical *Enterococcus faecalis* isolate. *Frontiers in Microbiology*.

[13] CLSI (2021). *Performance Standards for Antimicrobial Susceptibility Testing; Twenty-Fifth Informational Supplement*.

[14] Fiore E., Van Tyne D., Gilmore M. S. (2019). Pathogenicity of Enterococci. *Microbiology Spectrum*.

[15] Kristich C. J., Rice L. B., Arias C. A. (2014). Enterococcal infection—treatment and antibiotic resistance. *Enterococci: From Commensals to Leading Causes of Drug Resistant Infection*.

[16] Sattari-Maraji A., Jabalameli F., Node Farahani N., Beigverdi R., Emaneini M. (2019). Antimicrobial resistance pattern, virulence determinants and molecular analysis of *Enterococcus faecium* Isolated from children infections in Iran. *BMC Microbiology*.

[17] Arbabi L., Boustanshenas M., Rahbar M. (2016). Antibiotic susceptibility pattern and virulence genes in Enterococcus spp. isolated from clinical samples of Milad Hospital of Tehran, Iran. *Archives of Clinical Infectious Diseases*.

[18] Castillo-Rojas G., Mazari-Hiríart M., Ponce de León S., Mark Ibekwe A. (2013). Comparison of *Enterococcus faecium* and *Enterococcus faecalis* strains isolated from water and clinical samples: antimicrobial susceptibility and genetic relationships. *PLoS ONE*.

[19] Georges M., Odoyo E., Matano D. (2022). Determination of *Enterococcus faecalis* and *Enterococcus faecium* antimicrobial resistance and virulence factors and their association with clinical and demographic factors in Kenya. *Journal of Pathogens*.

[20] Maleki D., Manouchehrifar M., Kheljan M. N. (2021). Vancomycin-resistant Enterococcus Species: antimicrobial resistance and virulence genes profile. *Gene Reports*.

[21] Arfaatabar M., Shahbazi T., Ebrahimi T. (2022). Prevalence of vancomycin and gentamycin resistance among Enterococci spp. in Iran during 2007–2019: a systematic review. *Infection, Epidemiology and Microbiology*.

[22] Boccella M., Santella B., Pagliano P. (2021). Prevalence and antimicrobial resistance of Enterococcus Species: a retrospective cohort study in Italy. *Antibiotics*.

[23] Miller W. R., Munita J. M., Arias C. A. (2014). Mechanisms of antibiotic resistance in enterococci. *Expert Review of Anti-Infective Therapy*.

[24] Markwart R., Willrich N., Haller S. (2019). The rise in vancomycin-resistant *Enterococcus faecium* in Germany: data from the german antimicrobial resistance surveillance (Ars). *Antimicrobial Resistance & Infection Control*.

[25] Mengesha T. H., Ali M. M., Mengistu M., Assegu Fenta D., Falkinham Joseph (2024). High gastrointestinal colonization rate of vancomycin-resistant Enterococci among hospitalized patients: potential source for resistant gene. *International Journal of Microbiology*.

[26] Mundy L. M., Sahm D. F., Gilmore M. (2000). Relationships between Enterococcal virulence and antimicrobial resistance. *Clinical Microbiology Reviews*.

[27] Sparo M., Delpech G., García Allende N. (2018). Impact on public health of the spread of high-level resistance to gentamicin and vancomycin in Enterococci. *Frontiers in Microbiology*.

[28] Arias C. A., Contreras G. A., Murray B. E. (2010). Management of multidrug-resistant enterococcal infections. *Clinical Microbiology and Infection*.

[29] Pourakbari B., Aghdam M. K., Mahmoudi S. (2012). High frequency of vancomycin-resistant *Enterococcus faecalis* in an Iranian referral children medical hospital. *Maedica*.

[30] Sabouni F., Movahedi Z., Mahmoudi S., Pourakbari B., Keshavarz Valian S., Mamishi S. (2016). High frequency of vancomycin resistant *Enterococcus faecalis* in children: an alarming concern. *Journal of Preventive Medicine and Hygiene*.

[31] Kraszewska Z., Skowron K., Kwiecińska-Piróg J. (2022). Antibiotic resistance of Enterococcus spp. isolated from the urine of patients hospitalized in the university hospital in North-Central Poland, 2016–2021. *Antibiotics*.

[32] Hollenbeck B. L., Rice L. B. (2012). Intrinsic and acquired resistance mechanisms in Enterococcus. *Virulence*.

[33] Karna A., Baral R., Khanal B. (2019). Characterization of clinical isolates of enterococci with special reference to glycopeptide susceptibility at a tertiary care center of eastern Nepal. *International Journal of Microbiology*.

[34] Werner G., Klare I., Fleige C. (2012). Vancomycin-resistant VanB-Type *Enterococcus faecium* isolates expressing varying levels of vancomycin resistance and being highly prevalent among neonatal patients in a single ICU. *Antimicrobial Resistance and Infection Control*.

[35] Falgenhauer L., Preuser I., Imirzalioglu C. (2021). Changing epidemiology of vancomycin-resistant *Enterococcus faecium*: results of a genome-based study at a regional neurological acute hospital with intensive care and early rehabilitation treatment. *Infection Prevention in Practice*.

[36] Hayakawa K., Marchaim D., Palla M. (2013). Epidemiology of vancomycin-resistant *Enterococcus faecalis*: a case-case-control study. *Antimicrobial Agents and Chemotherapy*.

[37] Adams D. J., Eberly M. D., Goudie A., Nylund C. M. (2016). Rising vancomycin-resistant enterococcus infections in hospitalized Children in the united states. *Hospital Pediatrics*.

[38] Sivaradjy M., Gunalan A., Priyadarshi K., Madigubba H.s, Rajshekar D., Sastry A. S. (2021). Increasing trend of vancomycin-resistant Enterococci bacteremia in a tertiary care hospital, South India: A three-year prospective study. *Indian Journal of Critical Care Medicine*.

[39] Janjusevic A., Cirkovic I., Minic R. (2022). Predictors of vancomycin-resistant Enterococcus spp. intestinal carriage among high-risk patients in university hospitals in Serbia. *Antibiotics*.

[40] Hemapanpairoa J., Changpradub D., Thunyaharn S., Santimaleeworagun W. (2021). Does vancomycin resistance increase mortality? clinical outcomes and predictive factors for mortality in patients with *Enterococcus faecium* infections. *Antibiotics*.

[41] Sugai M., Yuasa A., Miller R. L. (2023). An economic evaluation estimating the clinical and economic burden of increased vancomycin-resistant *Enterococcus faecium* infection incidence in Japan. *Infectious Diseases and Therapy*.

[42] Emaneini M., Khoramian B., Jabalameli F. (2016). Prevalence of high-level gentamicin-resistant *Enterococcus faecalis* and *Enterococcus faecium* in an Iranian hospital. *Journal of Preventive Medicine and Hygiene*.

[43] Adhikari L. (2010). High-level aminoglycoside resistance and reduced susceptibility to vancomycin in nosocomial Enterococci. *Journal of Global Infectious Diseases*.

[44] Dadfarma N., Imani Fooladi A. A., Oskoui M., Mahmoodzadeh Hosseini H. (2013). High level of gentamicin resistance (HLGR) among Enterococcus strains isolated from clinical specimens. *Journal of Infection and Public Health*.

[45] El-Mahdy R., Mostafa A., El-Kannishy G. (2018). High level aminoglycoside resistant Enterococci in hospital-acquired urinary tract infections in Mansoura, Egypt. *Germs*.

[46] Labibzadeh M., Kaydani G. A., Savari M., Ekrami A. (2018). Emergence of high-level gentamicin resistance among Enterococci clinical isolates from burn patients in South-West of Iran: vancomycin still working. *Polish Journal of Microbiology*.

[47] Doss Susai backiam A., Duraisamy S., Karuppaiya P. (2023). Antibiotic susceptibility patterns and virulence-associated factors of vancomycin-resistant Enterococcal isolates from tertiary care hospitals. *Antibiotics*.

[48] Kibwana U. O., Manyahi J., Moyo S. J. (2024). Antimicrobial resistance profile of enterococcus species and molecular characterization of vancomycin resistant *Enterococcus faecium* from the fecal samples of newly diagnosed adult HIV Patients in Dar Es Salaam, Tanzania. *Frontiers in Tropical Diseases*.

[49] O’Driscoll T., Crank C. W. (2015). Vancomycin-resistant enterococcal infections: epidemiology, clinical manifestations, and optimal management. *Infection and Drug Resistance*.

[50] Misiakou M. A., Hertz F. B., Schønning K., Häussler S., Nielsen K. L. (2023). Emergence of linezolid-resistant *Enterococcus faecium* in a tertiary hospital in copenhagen. *Microbial Genomics*.

[51] Armin S., Zahedani S. S., Rahbar M., Azimi L. (2021). Prevalence and resistance profiles of vancomycin-resistant Enterococcal isolates in Iran; an eight-month report from nine major cities. *Infectious Disorders-Drug Targets*.

[52] Mobarez A., Doust R., Satari M., Teymornejad O., Yasliani S. (2009). Linezolid vancomycin resistant Enterococcus isolated from clinical samples in tehran hospitals. *Indian Journal of Medical Sciences*.

[53] Zaheer R., Cook S. R., Barbieri R. (2020). Surveillance of Enterococcus spp. reveals distinct species and antimicrobial resistance diversity across a one-health continuum. *Scientific Reports*.

[54] Aktas G. (2020). In Vitro efficacy of vancomycin combined with fosfomycin against vancomycin-resistant enterococci strains. *Pakistan Journal of Medical Sciences*.

[55] Farsi S., Salama I., Escalante-Alderete E., Cervantes J. (2023). Multidrug-resistant enterococcal infection in surgical patients, what surgeons need to know. *Microorganisms*.

[56] Olearo F., Both A., Belmar Campos C. (2021). Emergence of linezolid-resistance in vancomycin-resistant *Enterococcus faecium* ST117 associated with increased linezolid-consumption. *International Journal of Medical Microbiology*.

